# Using natural experiments to improve public health evidence: a review of context and utility for obesity prevention

**DOI:** 10.1186/s12961-020-00564-2

**Published:** 2020-05-18

**Authors:** Melanie Crane, Erika Bohn-Goldbaum, Anne Grunseit, Adrian Bauman

**Affiliations:** The Australian Prevention Partnership Centre, Sydney School of Public Health, Charles Perkins Centre, The University of Sydney, Camperdown, NSW Australia

**Keywords:** Natural experiments, obesity prevention, evaluation methods, study design, physical activity, nutrition, population health interventions, narrative review

## Abstract

**Background:**

Natural experiments are increasingly valued as a way to assess the health impact of health and non-health interventions when planned controlled experimental research designs may be infeasible or inappropriate to implement. This study sought to investigate the value of natural experiments by exploring how they have been used in practice. The study focused on obesity prevention research as one complex programme area for applying natural experiment studies.

**Methods:**

A literature search sought obesity prevention research from January 1997 to December 2017 and identified 46 population health studies that self-described as a natural experiment.

**Results:**

The majority of studies identified were published in the last 5 years, illustrating a more recent adoption of such opportunities. The majority of studies were evaluations of the impact of policies (*n* = 19), such as assessing changes to food labelling, food advertising or taxation on diet and obesity outcomes, or were built environment interventions (*n* = 17), such as the impact of built infrastructure on physical activity or access to healthy food. Research designs included quasi-experimental, pre-experimental and non-experimental methods. Few studies applied rigorous research designs to establish stronger causal inference, such as multiple pre/post measures, time series designs or comparison of change against an unexposed group. In general, researchers employed techniques to enhance the study utility but often were limited in the use of more rigorous study designs by ethical considerations and/or the particular context of the intervention.

**Conclusion:**

Greater recognition of the utility and versatility of natural experiments in generating evidence for complex health issues like obesity prevention is needed. This review suggests that natural experiments may be underutilised as an approach for providing evidence of the effects of interventions, particularly for evaluating health outcomes of interventions when unexpected opportunities to gather evidence arise.

## Background

Many public health issues are complex, requiring preventive health actions targeted at multiple upstream social and environmental determinates to improve population-level outcomes [1]. Despite this, the published literature is almost entirely focused on short-term individual-level research outcomes and lacking complex, multi-level, population-level intervention evidence [2]. Obesity is now recognised as a complex health issue, driven by multiple interrelated factors, including environmental, social and cultural determinants beyond individual-level determinants of behaviour [3–5]. Today, over 700 million adults and children are obese [6]. Effective population-wide prevention strategies implemented at-scale are needed to combat obesity; individually targeted strategies, such as health education and behavioural skills, have largely been found to be ineffective and unsustainable [2, 7]. Recommended prevention strategies focus on environmental interventions and policies to promote healthy eating and physical activity, such as taxation and restrictions on advertising of unhealthy food, interventions to increase healthy food availability, and environmental changes to the built environment [8]. However, the effectiveness of these interventions or policies remains limited by a lack of evaluation evidence.

Public Health evidence has been somewhat restricted to individualised prevention and treatment interventions from randomised controlled trial (RCT) studies. The key challenges for assessing complex policy and environmental interventions is that RCT studies are rarely appropriate, or even possible, in most situations [9]. In these complex interventions, the intervention is unlikely to be investigator initiated and the researcher is unlikely to have direct control over the study environment or wider policy influences [10, 11]. Other factors related to the complexity of populations and context make it unrealistic to apply controlled study environments such as the long time over which health behaviours change or outcomes are established [12, 13]. These issues have led to calls for natural experiment studies to improve the evidence base for public health interventions [11, 14].

The Medical Research Council (MRC) in the United Kingdom describes natural experiments as evaluating health or other outcomes where “*exposure to the event of intention of interest has not been manipulated by the researcher*” [15]. A number of other definitions exists and contributes to widespread confusion that a natural experiment is a type of study design (synonymous with or distinct from a quasi-experiment) rather than what is usually an unplanned ‘opportunity’ for research [15]. There is also some debate as to whether a natural experiment should refer only to studies which are ‘experimental’, i.e. where variation in the outcomes are analysed using methods that attempt to make causal inference. This position is held by the MRC and would include quasi-experimental studies but would exclude observational study designs as insufficient for determining causality [16]. Others contest that even weak study designs may be better than no evidence at all [17, 18]. Hence, in evaluating the contribution of natural experiment studies to the evidence base, it is important to consider both the strength of their designs and their potential value to the existing evidence base.

The purpose of this paper is two-fold: firstly, given the increasing advocacy for using natural experiments, we sought to investigate how natural experiments have been defined and used in practice. Specifically, we describe and assess the characteristics of natural experiments conducted in the area of obesity prevention to reveal the strengths, gaps, and weaknesses and help inform future research practice. Secondly, we explore the value of natural experiments in evaluating real-world interventions by considering the extent to which a planned experiment might be possible or whether this knowledge could only have been generated by a natural experiment.

## Methods

### Literature search strategy

A literature review was conducted on published peer-reviewed studies that self-described as natural experiments and focused on obesity prevention through improving nutrition or physical activity. The purpose of the review was to explore the use and utility of natural experiments [19], not to determine the outcome effects of interventions, which has been reported elsewhere [20, 21]. A systematic search in Scopus, Web of Science and CINAHL databases identified potential studies by combining two main topics (natural experiment AND population health) with the areas of interest: physical activity, nutrition or obesity (for full search criteria details, see Additional file S1). The search results were then limited by language (English only), article type (original research published/in-press and full-articles only), and dated from 1 January 1997 to 22 December 2017. Articles involving non-human research or not related to obesity prevention or improving nutrition or physical activity were excluded.

**Fig. 1:**
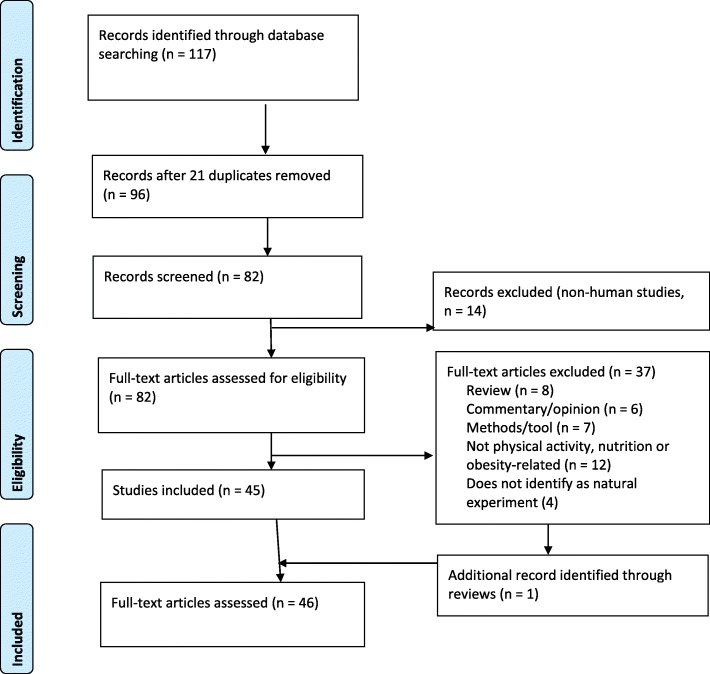
Study selection

Figure 1 depicts the study selection stages. The initial search results were combined (*n* = 117 articles) and duplicate studies (*n* = 21 articles) were removed. Titles were scanned and 14 additional articles were removed as non-human studies, resulting in 82 articles. Two authors (MC and EG) read the abstracts to confirm that the articles met the eligibility criteria. A further 37 studies were removed as a commentary or opinion piece (*n* = 6), a protocol or methods article (*n* = 7), a review or meta-analysis (*n* = 8), or unrelated to obesity prevention (*n* = 12); an additional 4 four studies were removed as they did not self-identify as natural experiments (Additional file S2). The reference lists of the review articles were also searched for natural experiment studies contained within and 1 additional study was identified. The full text of all 46 articles was evaluated to confirm all inclusion criteria were met.

### Critical review of studies

To investigate use, identified studies were described according to the nature of the intervention (type/setting, theme and scale) and of the evaluation methodology (study design, the exposure, characteristics of the exposed group and inclusion of a control group, and data collection measures and period). We then conducted a qualitative synthesis on the value of the studies to the field (Box 1). This includes consideration of the purpose of the study, and whether it could be answered from a planned, researcher-driven study, following the typologies developed by Dawson and Sim [22]. We discuss whether a planned controlled experiment (specifically an RCT or cluster RCT) would be realistic, considering practical issues such as time and population constraints, the potential for ethical conflict, and other limitations of RCTs [12, 13, 23].

MC and EG independently assessed each of the included studies and met to identify and resolve any coding and data extraction discrepancies. Where the authors disagreed, the other authors were consulted and a consensus reached.

## Results

The literature search resulted in 46 studies directly related to the topic of diet, physical activity and obesity prevention, and are described in Table 1. Among the natural experiments conducted in this area over the last 20 years, none were published before 2007, 7 were published prior to 2012 (when the MRC guidance was published) and 4 more were published the same year. The largest annual publication volume occurred in 2017 with 14 published studies, at least double the numbers of any previous year (Additional file S3). The studies were predominantly from North America (22 from the United States of America and 6 from Canada). The remaining studies were from Europe (*n* = 7), Australia (*n* = 8) and South America (*n* = 3).

**Table 1:** Studies self-identifying as natural experiments which met inclusion criteria

Author, year	Geography	Type	Exposure	Exposed group	Unexposed group	Intervention outcome	Study design	Primary data
Allais et al., 2015 [27]	France	P	Food labelling	Consumers of supermarket stores	No control group (pre exposure)	Sale of healthy food items	Interrupted time series	No
Barlow et al., 2017 [28]	Canada	P	Tariff/trade agreement	Residents in country with tax on sugar sweeteners	Residents in countries with no tax on sugar sweeteners	Sales of sugar foods/beverage	Pre/post cohort + control	No
Barradas et al., 2017 [29]	Colombia	C	PA programme (park based)	Residents near park with PA programme	Residents near parks without programme; with existing programme	Quality of life	Post only	Yes
Bernatchez et al., 2015 [30]	Canada	E	Bike share programme	City residents near bike-share docks	City residents further from bike-share docks	Programme awareness	Repeated cross-sectional	Yes
Brandt et al., 2017 [31]	United States	P	Food regulation	Residents in counties with TFA restrictions	Residents in counties with no TFA restrictions	Hospital admission for cardiovascular events	Pre/post cohort + control	No
Cawley et al., 2007 [32]	United States	P	PE education	Students in high schools with PE requirements	Students in high schools without PE requirements	PA, weight	Post only	No
Cawley et al., 2010 [33]	United States	P	Social welfare benefit	Elderly receiving inflated social security benefits	No control group (variation in exposure)	Weight	Repeated cross-sectional	No
Christian et al., 2013 [34]	Australia	E	Built environment (housing relocation)	Residents in a PA-supportive neighbourhood	residents in conventional or hybrid neighbourhood	Walking	Pre/post cohort + control	Yes
Christian et al., 2017 [35]	Australia	E	Built environment (housing relocation)	Residents in a new neighbourhood	No control group	Walking	Pre/post cohort	Yes
Cohen et al., 2012 [36]	United States	E	Green space (outdoor gym equipment)	Park users in parks with new fitness equipment	Park users in parks without equipment	PA METs	Pre/post cohort + control	Yes
Cornelsen et al., 2017 [37]	United Kingdom	P	Tax (sugar)	Customers of restaurant chain	No control group (pre exposure)	Sugar-sweetened beverage sales	Interrupted time series	No
Cranney et al., 2016 [38]	Australia	E	Green space (outdoor gym equipment)	Park users in parks with new fitness equipment	No control group (pre exposure)	PA	Repeated cross-sectional; qualitative interviews	Yes
Datar & Nicosia, 2017 [39]	United States	P	Restricted access unhealthy food	Children living in states with school policies on discretionary foods	Children in states without policies	BMI	Post only	No
Dill et al., 2014 [40]	United States	E	Built environment (bicycle infrastructure)	Residents in streets with bike paths	Residents near streets without paths	PA/AT	Pre/post cohort + control	Yes
Dubowitz et al., 2015 [41]	United States	E	Food environment (access to healthy food outlet)	Residents in neighbourhoods with no full-service supermarket	No control group (baseline data only)	Healthy food purchases	Pre only	Yes
Elbel et al., 2011 [42]	United States	P	Food labelling	Consumers (low-income kids/teens) of fast-food outlets in city with mandatory food labelling	Consumers (low-income kids/teens) of fast-food outlets in city with no food labelling	Food consumption - fast food	Pre/post cohort + control	Yes
Ferguson et al., 2017 [43]	Australia	p	Food affordability (pricing)	Residents in remote Aboriginal communities	No control group (variation in exposure)	F&V, general food/beverage sales	Interrupted time series; repeat cross-sectional; Qualitative interviews	Mixed
Frew et al., 2014 [44]	United Kingdom	C	PA programme (access to services)	City residents with limited access to free PA services	Hypothetical (as if there was no free access)	PA-related diseases, QALYS	Pre/post cohort	Yes
Fu & VanLandingham, 2012 [45]	United States	O	Migration	Vietnamese immigrants in America	Vietnamese immigrant returnees and native (never-leavers)	BMI, waist-hip circumference	Post only	Yes
Gee et al., 2015 [46]	United States	O	Migration	Filipinos in America	Native Filipinos	BMI, waist-hip circumference	Pre/post cohort + control	Yes
Giles-Corti et al., 2013 [47]	Australia	E	Built environment (housing relocation)	Residents in a PA-supportive neighbourhood	residents in conventional or hybrid neighbourhood	AT and recreational PA	Pre/post cohort + control	Yes
Hobin et al., 2017 [48]	Canada	P	Food labelling	Consumers of supermarkets with nutrition labelling	Consumers of supermarkets without nutrition labelling	Sale of healthy food items	Pre/post cohort + control; qualitative interviews	Mixed
Jancey et al., 2016 [49]	Australia	E	Built environment (workplaces)	Office workers move to new building	No control group (pre exposure)	PA	Repeated cross-sectional	Yes
Jones-Smith et al., 2017 [50]	United States	O	Economic resources (casino developments)	Native American new-borns born in area near casinos (greater resources)	Native American new-borns near no casino (lower resources)	Birth weight (childhood obesity)	Repeated cross-sectional	No
Jürges et al., 2011 [51]	Germany	P	Education reform (years educated)	Country population years of education	No control (variation in exposure)	Overweight, obesity, smoking	Repeated cross-sectional	No
Kapinos et al., 2017 [52]	United States	O	Clinical (fetal mal-presentation at birth delivery)	Mothers who deliver by caesarean birth	Mothers who deliver by vaginal birth	Mothers’ postpartum BMI	Pre/post cohort + control	No
Kesten et al., 2014 [53]	United Kingdom	E	Built environment (AT infrastructure)	Commuters in neighbourhoods proximal to AT infrastructure	No control group (pre exposure)	Awareness of AT infrastructure	Qualitative interviews; media	Mixed
Kodish et al., 2016 [54]	United States	O	Economic resources (casino ownership)	Native American tribal communities with casino ownership profit	Tribal communities with little/no cash profit	Access to healthy food, PA facilities, etc.	Qualitative interviews	Yes
Lee et al., 2017 [55]	United States	E	Built environment (transfer to local school)	Students transferred to local school	No control group (recall pre exposure)	AT participation	Post only	Yes
Madsen, 2011 [56]	United States	P	Clinical (health (BMI) screening optional notification)	Students in schools with screening-notified parents	Students in schools with screening not notified	BMI	Repeated cross-sectional; qualitative interviews	No
Odoms-Young et al., 2014 [57]	United States	P	Food access (change to nutritional content of welfare food package)	Low-income families receiving food security packages	No control group (pre exposure)	Dietary intake, Body weight	Pre/post cohort	Yes
Ovrum et al., 2014 [58]	Norway	P	Food access (healthy food)	Families of children whose schools give free F&V	Families of children who do not have F&V programme or have optional programme	F&V consumption	Post only	Yes
Pollack et al., 2014 [59]	United States	P	Social housing	Public housing residents in scattered housing	Public housing residents in clustered housing	Diet, PA behaviour, BMI, perceived health	Post only	Yes
Ram et al., 2016 [60]	United Kingdom	E	Social housing	City residents from different housing sectors	No control group (baseline data only)	PA, BMI, self-reported health	Pre only	Yes
Sadler et al., 2013 [61]	United States	E	Food environment (access to healthy food outlet)	Residents in neighbourhoods with new supermarket	Residents in neighbourhood with no supermarket	F&V intake, food security	Pre/post cohort + control	Yes
Schultz et al., 2017 [62]	United States	E	Built environment (street redesign)	Low-income neighbourhood residents	No control group (pre exposure)	PA, park usage	Repeated cross-sectional	Yes
Simões et al., 2017 [63]	Brazil	C	PA programme (community)	Cities participating in the programme	Non-participatory and not-yet-participatory cities	PA	Pre/post cohort + control (step wedge)	Yes
Stanley et al., 2016 [64]	Canada	E	Public transport strike	University students using public transport and other transport modes	No control group (pre-exposure)	PA, AT participation	Pre/post cohort	Mixed
Stone et al., 2012 [65]	Canada	P	PA programme (school curriculum)	Primary students in school with daily PA policy (mandatory minimum PA minutes)	Primary students in school without PA curriculum	PA, BMI	Post only	Yes
Sutherland et al., 2010 [66]	United States	P	Food labelling	Consumers of supermarkets with shelf nutrition labelling	No control group (variation in exposure)	Sale of healthy food items	Interrupted time series	No
Torres et al., 2017 [67]	Colombia	C	PA programme (park-based)	Residents near park with new PA programme	Residents near parks without programme or with established programme	MVPA	Pre/post cohort + control	Yes
Tudor-Locke et al., 2008 [68]	Australia	E	Built environment (housing relocation)	Residents relocating home within a metropolitan area	No control group (pre exposure)	PA	Pre/post cohort	Yes
Veitch et al., 2012 [69]	Australia	E	Green space	Users of a refurbished park	Users an un-furbished park	PA, park usage	Pre/post cohort + control	Yes
Wagner et al., 2013 [70]	United States	C	Social living arrangements	Student at a university	No control group (variation in exposure)	Various health behaviours (PA; alcohol)	Post only	No
Watson et al., 2016 [71]	Canada	P	Economic (change in unemployment benefits)	Adult workforce population	No control group (variation in exposure)	Obesity	Pre/post cohort	No
Zick, 2014 [72]	United States	P	Daylight savings scheme	Residents in states with daylight savings	Residents in states with no daylight savings	MVPA	Pre/post cohort + control	No

*AT* active travel, *BMI* body mass index, *C* community intervention, *E* environmental intervention, *F&V* fruit and vegetable intake, *METs* metabolic equivalent expenditures, *MVPA* moderate-to-vigorous physical activity, *O* other, *P* policy, *PA* physical activity, *PE* physical education, *QALYs* quality-adjusted life years, *TFA* trans-fatty acid

### Intervention context

#### Setting and scale

The majority of the natural experiments identified were policy interventions [27, 28, 31, 32, 37, 39, 42, 43, 48, 51, 56, 58, 59, 65, 66, 71, 72], followed by environmental interventions [30, 34–36, 38, 40, 41, 47, 49, 53, 55, 60–62, 64, 68, 69] and community-based interventions [29, 44, 63, 67, 70]. Fewer studies were of economic interventions [33, 50, 54], individual behaviour interventions [52] and anthropological studies [45, 46] (Table 2). In terms of the scale of implementation, the majority of interventions were conducted at the neighbourhood scale. All but two [31, 42] of the 19 policy interventions were implemented at a national or state level.

**Table 2:** Summary of study characteristics

Classification	Sub-classification	N
**Intervention characteristics**		46
Intervention setting	Policy intervention	19
	Environmental intervention	17
	Community intervention	5
	Individual behaviour intervention	1
	Economic intervention	2
	Migration	2
Scale of intervention	National	8
	State/region	12
	City/neighbourhood	26
Intervention theme	Food labelling	4
	Food regulation/taxation	3
	Food accessibility	6
	Workplace/school/community physical activity programme	7
	Built infrastructure development	11
	Transport interventions	4
	Social environment changes	3
	Clinical/screening procedure	2
	Economic events	4
	Other	2
**Evaluation characteristics**		
Study design	Longitudinal pre/post quasi-experiment	15
	Longitudinal pre/post cohort (single group)	6
	Interrupted time series	4
	Repeat cross-sectional surveys	9
	Post-test observation only	9
	Qualitative only	2
	Pre-test observation only	2
Data source	Primary	27
	Secondary	15
	Mixed	4
Data collection^a^	Short-term (<3 years)	21
	Long-term (3+ years)	12

^a^Includes time-related study designs only

#### Intervention theme

The natural experiments related to diet assessed exposure to the introduction of, or changes to, food labelling [27, 42, 48, 66], or food regulation and taxation [28, 31, 37], and access to, or affordability of, healthy food options such as fruit and vegetables [39, 41, 43, 57, 58, 61]. Natural experiments related to physical activity included exposure to workplace physical activity programmes [49], schools [32, 65], park settings [29, 67], community-wide programmes [30, 44, 63], and built environment interventions, including infrastructure for active travel and changes in street design [30, 40, 53, 62], residential/school development [34, 35, 47, 55, 59, 60, 68] and green spaces [36, 38, 69]. Other experiments were conducted to assess physical activity outcomes associated with disruption to transport services [64], daylight saving [72] and social habitat [70]. Obesity-related outcomes more broadly were evaluated in relation to exposure to clinical procedures [52, 56], migration [45, 46], education [51] or economic events [33, 50, 54, 71].

### Evaluation characteristics

#### Study design

A variety of study designs were used, with several quasi-experiments, comparing longitudinal pre/post intervention changes in exposure of a cohort against a control or comparison group [28, 31, 34, 36, 40, 42, 47, 48, 52, 61, 63, 67, 69, 72]. The remaining cohort studies were observational (non-experimental), comparing pre/post exposure without a control group [35, 44, 57, 64, 68, 71]. Four studies conducted interrupted time-series on sales data [27, 37, 43, 66], two without a control comparator (observational only) [43, 66]. Observational studies included repeat cross-sectional surveys [30, 33, 38, 43, 49–51, 56, 62], some of which incorporated mixed methods (qualitative interviews or geospatial mapping) [38, 43, 48, 56], or used a cross-sectional single time point only [29, 32, 39, 41, 45, 55, 58–60, 65, 70]. One study pooled repeat cross-sectional data as a result of data availability and was therefore unable to assess time-related effects [32]; another [46] was a feasibility study anticipating a larger, longitudinal investigation. Two studies were qualitative only [53, 54].

#### Data source

Natural experiment studies used a variety of sources. In total, four studies collected both primary and secondary data [43, 48, 53, 64] and 27 studies only collected primary data. Fifteen studies used secondary routinely collected surveillance data from national or state censes and health surveys [31–33, 51, 56, 71, 72], hospital administrative data [50, 52], pre-existing cohort studies [39, 70] and food supply [28] or sales data [27, 37, 43, 66]. Three studies used simulated data to establish a hypothetical unexposed group [27, 28, 44].

#### Exposure

Exposure samples comprised residential populations in a defined neighbourhood or region, consumers of food outlets (e.g. supermarkets or restaurant chain) or physical activity space (e.g. park-setting). A clearly defined unexposed ‘control’ group was used by 15 studies [28, 31, 34–36, 40, 42, 46–48, 52, 61, 63, 67, 69, 72]. More often, exposure was assessed in the one population group or area without a defined unexposed group (*n* = 18) [27, 30, 33, 38, 43, 44, 47, 49–52, 56, 57, 62, 64, 68, 70, 71] or comparisons made between two groups, one of which was exposed, at a single time point (*n* = 10) [29, 32, 39, 41, 45, 55, 58–60, 65].

Not all studies used a dichotomous definition of exposure. Graded levels of exposure between groups, areas or individuals over time were used in a small number of studies [33, 43, 51, 71]. These studies had no similar intervention characteristics that might suggest a pattern or typology of where and when graded exposure may be necessary.

#### Evaluation measures

A variety of evaluation measures were used to evaluate obesity prevention interventions, including subjective and objective measures of physical activity (walking, active travel, metabolic equivalents), diet and body mass index, and health behaviour from surveys, physical and manual counters (i.e. accelerometers), as well as secondary data sources listed above. One study used geospatial information systems to identify exposed and unexposed groups [35].

Evaluation methods varied across similar interventions. For example, two studies [36, 38] evaluated the impact of new outdoor gym equipment on physical activity. Only one compared changes in the exposed group (park users/residents) against users of parks without gym equipment. Three studies used different methods to assess sales data to determine the effectiveness of food labelling policies in supermarkets. One of these compared data across three supermarket chains, with one chain acting as the control [55], one compared variation in exposure across a variety of supermarket types [66], and the third study simulated exposure because the labelling policy in question was voluntary and haphazardly implemented [27]. These differences suggest a variety of evaluation measures are applied to natural experiment opportunities.

#### Evaluation timeframe

Of the studies that included pre and post data collection, studies conducted over a short (less than 3 year) evaluation period, such as community or environmental interventions, predominantly used original sourced data from surveys or observational measures. Studies with a longer evaluation period relied on secondary data collection as could be expected given the time constraints on primary data collection.

### Utility

The value of natural experiments for providing real-world population evaluation evidence was appraised in terms of whether the studies could have been conducted as planned researcher-driven experiments. We determined the purpose of the study and found that the majority evaluated the effectiveness of an intervention or impact of a policy (see Additional file 4 Table S2 for full list). In terms of utility, we first found that planned researcher-driven research was not always feasible to answer the research questions raised by the authors and, as such, were opportunities that could only ever be investigated as natural experiments. For example, Jones-Smith et al. [50] sought to establish whether Native American economic resources (from casino ownership) influenced the likelihood of childhood obesity; Zick et al. [72] aimed to assess whether daylight saving time is associated with increased time spent in moderate-to-vigorous physical activity. The purpose of these studies was to examine social or environmental determinants or inequalities in the population, which would not be amenable to/appropriate for researcher manipulation. In some instances, we recognised it would be possible for a researcher to have some involvement in the decisions regarding when and where an intervention occurs in order to obtain baseline data; for example, evaluation of the impact of a new policy taxation [27, 37]. However, if a policy was enacted quickly, baseline data collection would be restricted. Some of the studies involved relocation, such as those for new housing developments in Australia [34, 35, 47, 68], where researcher control over the planning, timing or conditions of the relocation, even if working with the authorities, would likely be unrealistic.

We note that studies aimed at assessing inequalities in the population or determining intervention effectiveness would be unethical as a planned experiment where there was risk of potential harm from intentionally restricting access to medical care [52], economic support [50], educational opportunity [51] or randomising individuals/groups to social benefits [33, 57, 71], freedom to migrate [45, 46] or health services [57, 65].

In other cases, researcher manipulation of study components may have been possible, but the researcher would have been constrained by practical considerations such as time, population sample size or location of exposure [61]. As obesity takes time to develop and tends to reverse, some evaluation questions necessitate a long lag time between exposure and outcome, which negate short-time planning. In one study, historical data was used to investigate the effect of academic schooling on obesity-related health behaviours in adults [51]. A planned study of this nature would be unrealistic, involving long follow-up from schooling to adult years to establish causality; it would also be unethical to control exposure to schooling. Bernatchez et al. [30] evaluates awareness of a new bicycle share programme rather than use; this may be because evaluation occurred too early to measure behaviour change as measured in another similar study [40]. In other cases, a planned experiment would be unpractical because the nature of the intervention necessitates a whole-of-population approach, such as the effect of tariffs applied to certain energy dense foods on unhealthy food consumption [28]. The point of time at which a researcher engages may preclude pre-intervention data collection. For example, in one study, researchers could only collect retrospective survey data on commute mode from parents whose children transferred to a new school [55]; other researchers serendipitously had a public transit strike occur in the midst of measuring spatial behaviour among undergraduate students, allowing a spontaneous pre/post examination on changes to student travel patterns to university [64]. In some situations, a natural experiment may be the only realistic option available despite the absence of a control group because the intervention is so unique that a suitable control group is not feasible. For example, in the study by Cranney et al. [38], the park setting in which equipment was installed may have had unique features specific to its local environment, precluding a suitable control. Similarly, in the study by Barradas et al. [29], some parks were already receiving the intervention programme and intervention settings were pre-determined by another body.

## Discussion

This study has characterised the use and value of natural experiments, particularly focusing on the area of obesity prevention and its complex aetiology. Although the number of studies self-described as natural experiments has increased over the past two decades, the body of research, at least in obesity prevention, remains small.

Obesity prevention is a complex issue. Research evidence about interventions implemented in real-world conditions and the impact of policies represent a key gap in the knowledge. Thus, there is a need for greater generation of evidence about the impact and effectiveness of policy strategies, and natural experiments could be better utilised to provide this evidence. In assessing how natural experiment studies have been used, we found that the majority of the studies reviewed were designed to evaluate the impact of a policy or the effectiveness of an environmental intervention. The policy evaluation studies were almost all at the national or state level. Geographically, the studies in this review were located in English-speaking countries, which may reflect the high prevalence rates of obesity in these countries, and the search methods precluded studies in non-English speaking countries; however, it may also be due to the low use of evaluation approaches across other countries and within research groups within these countries. For this reason, WHO Europe recently held a workshop to support and facilitate public health practitioners from participating European countries in the use of natural experiment methods [14].

### Strengths and weaknesses of natural experiments

The strengths of natural experiments are in their ability to evaluate the process and outcomes of implementation of policies and interventions within the real-world complex social and political conditions they naturally operate in. The response to the obesity epidemic has required a broad range of policy, environmental and individual behaviour change interventions – necessarily complex interventions able to function within a complex socio-political system [2, 23]. Evaluation research designs need to be flexible and able to measure the interaction between multiple factors [73].

Natural experiments offer opportunistic evidence where a researcher-driven study may be impossible for reasons of intervention timing or exposure. Nevertheless, the ability to make causal inferences from natural experiments depends on optimising the research study design [74]. We identified a variety of designs, including interrupted time-series, cross-sectional and longitudinal cohort designs. Few of these studies were experimental/quasi-experimental, including both pre and post measures for an exposed and a comparator group. However, a surprising number of the studies used a single data collection point to evaluate an intervention and thus could not attribute any observed changes to exposure to the intervention. However, some used mixed methods designs to strengthen study findings. Two studies presented only baseline data (pre-experimental) [41, 60] and may have further intentions for collecting follow-up data; these may be premeditated natural experiments. This evidence represents a weakness in study design for evaluating natural experiment studies rather than a weakness of natural experiments in general, and something the MRC has tried to address through detailed guidance on measurement and statistical methodologies [26].

Another strength of natural experiments is their flexibility. Evaluation periods ranged from a single time point to spanning decades and unsurprisingly the evaluation period determined whether primary or secondary data was used to assess exposure. A variety of outcome measures was used and these sometimes varied across similar interventions. While this flexibility is an advantage of the method, it increases the difficulty for comparisons in evidence reviews [21]. Most studies adapted the research design to fit the existing intervention context. Harnessing resources before policy interventions is a research planning challenge posed by natural experiments. To overcome this, some studies strengthened their findings by the use of multiple data sources. In such situations, both primary and secondary data contribute to the evidence, and largely depend on the level of control the researcher has on the intervention occurring, the group exposed or the timeframe in which the intervention or event occurs.

A further strength of natural experiments is their delivery of scale, allowing for exploration of a wider range of research questions to be investigated at the population level, to enable generalisation [15]. Natural experiments operate under circumstances where randomisation is not possible, there are ethical considerations, identifying suitable controls may problematic, and timing (both researcher timing relative to the intervention and the time length of the intervention) may make the collection of ideal data infeasible. These natural experiments fill a void not otherwise met by traditional designs, and may yield insights into exposure–outcome relationships, which are nonetheless informative for obesity prevention.

Natural experiments are often criticised for their inability to eliminate bias. Benton et al. [75] assessed the risk of bias across a number of natural experiments using an acceptability score based on confounding, participant selection, exposure and outcome measures, and missing data, concluding that evidence on the effects of these interventions was too problematic to be useful. Bias, particularly due to confounding, is a concern for natural experiments [18]. We observed that few studies in this review had a defined control and exposed group – given that these studies are unplanned evaluations, a clearly defined control group may be challenging to establish. Within the studies reviewed, a clearly defined unexposed group was more likely in environmental interventions where the researcher could establish a study design with original data. In contrast, establishing a clearly defined unexposed group becomes more difficult when evaluating policy interventions, as these are generally implemented acutely, and at the population level. Modelling simulations to create control groups was one approach employed [27, 44] to circumnavigate the lack of control group comparator. Others employed a step-wedge design [63] or time-series data to help overcome some limitations of not having a suitable control comparator condition. Simulations may be especially useful when counterfactuals are difficult to establish or create [26]. Leatherdale [18] provides some other suggestions for facilitating greater evaluation evidence of health policies, suggesting the need to improve the ability to evaluate government policy. For this to happen, capacity needs to be built around practitioners either to conduct natural experiments or to work closely with academics so that more robust quasi-experimental methods of evaluation can be employed [76].

Despite these weaknesses, we caution against devaluing natural experiments based on a simple hierarchy of evidence. Applying the same standards of study design quality to natural experiments ignores the contribution they can make to overall evidence generation [17], particularly in regard to the complexity of real-world interventions and policy evidence. In response to the complexity of the obesity epidemic, obtaining policy and intervention impact evidence is critical. Indeed, natural experiments may provide innovative research translation evidence that has been lacking on obesity policy interventions [77, 78]. Therefore, despite the limitations of natural experiments, they provide valuable information on public health efforts to prevent obesity as, otherwise, any effects might remain unknown.

The limitations of this review include our search criteria for identifying studies. It is possible that the confusion around natural experiments and quasi-experiments limits the number of collected studies to those we have discussed. For example, some protocol papers extracted from the initial search identified as a natural experiment, yet subsequent articles by the authors of these papers did not self-identify their intervention as a natural experiment. This suggests that those studies that want to identify as this type of research are not meeting an agreed definition. The synthesis of the utility of studies was limited to the information provided in each article; political, socio-cultural and practical obstacles that affect natural experiments and limit the potential for planned interventions is thus speculative.

## Conclusion

This review examined natural experiments in an effort to improve the public health evidence for obesity prevention where a controlled experimental design would be inappropriate. Research using natural experiments has increased over the last few years; however, it remains overlooked in the context of the wider research evidence despite the importance of these interventions taking place in real-world settings. Our findings highlight the strength of natural experiments in improving our understanding of the effectiveness of complex population interventions and providing informed evidence of the impact of policies and novel approaches to understanding health determinants and inequalities. The studies reviewed also reveal that there is need to strengthen research designs to enhance their utility.

Box 1 Determining the utility of a natural experiment 1. Is there a need to generate population health research evidence about this issue? [24, 25]

Does it provide evidence on the impact or effectiveness of a policy or environmental intervention, address health inequalities in the population or how to change behaviour?

2. Is a planned researcher-controlled study possible? [22]

a. No control – no ability to control where the intervention occurs, whom it affects or when it starts or finishes

b. Partial control – ability to influence exposure timing or location (i.e. able to arrange with the relevant authorities to obtain comparative baseline data)

3. Is a planned researcher-controlled study ethical? [22, 26]

a. Creating the conditions for an experiment to determine this issue would be unethical (e.g. where it may create more harm)

b. Random assignment to the intervention would be unethical (e.g. withholding the only known cure for a disease)

4. Is a planned researcher-controlled study practical? [12, 13, 25]

c. Sufficient population available for randomisation: total number of planned exposed/unexposed clusters required would be unrealistic to achieve

d. Time for follow-up: unlikely for a researcher-driven study could accommodate the prolonged periods necessary for outcomes to establish across entire populations

e. Representativeness of the population: threat to applicability of a study if the population sample is too different from the population it was intended to represent

f. Relative costs: sufficient sample and time lag before health impacts evolve would be costly to implement

g. Programme complexity: the intervention is so complex that a controlled study environment would be infeasible

h. Intervention scale-up: an intervention is known to be efficacious/effective but requires demonstration of its effectiveness on a scale unlikely in a researcher-driven context

i. Real-world conditions: purpose is to document contextual factors constituting ‘real-world’ implementation conditions - experimental approach is antithetical to this purpose

## Supplementary information

**Additional file 1: Table S1.** Search strategy.

**Additional file 2: Table S2.** Describes studies excluded through abstract review.

**Additional file 3: Figure S1.** Showing the use of natural experiment studies surrounding nutrition and physical activity interventions on obesity.

**Additional file 4: Table S3.** Describes the research aims of included studies.

## Data Availability

Data generated or analysed during this study are included in this published article and its additional files. Please contact the corresponding author for any additional queries.

## Abbreviations

MRC: Medical Research Council
RCT: Randomised controlled trial
