# Supplementary material for: Using natural experiments to improve public health evidence: a review of context and utility for obesity prevention
Source: Health Res Policy Syst. 2020 May 18;18:48. doi: 10.1186/s12961-020-00564-2 (PMC7236508; doi:10.1186/s12961-020-00564-2)
Supplement: Supplementary file 1 — Additional file 1: Table S1. Search strategy. [file 12961_2020_564_MOESM1_ESM.docx]

## Additional file 1

### Search strategy

Literature searches were conducted 22 December 2017 in Scopus, Web of Science and Cinahl (all databases). The search included combining two main topics (natural experiment AND population health) with the areas of interest: physical activity, nutrition or obesity. The Boolean operator NEAR was used with the two main topic search terms as previous search strategies were either too broad with many irrelevant references or too narrow. Results were limited by language (English only) and publication date (1997 forward). Article types were restricted, excluding some (e.g., reviews) and limiting to others (articles published or in press), as able within each database. Web of Science was further limited to exclude the CABI and Zoological Record databases. The Scopus strategy is shown as the example, below, and the two other database searches are available by request.

### Scopus search

( ( TITLE-ABS-KEY ( physical AND activity OR physically AND active ) OR TITLE-ABS-KEY ( physical AND inactivity OR physically AND inactive ) OR TITLE-ABS-KEY ( sedentary ) OR TITLE-ABS-KEY ( food OR nutrition OR diet ) OR TITLE-ABS-KEY ( overweight OR obesity OR obese ) ) ) AND ( ( TITLE-ABS-KEY ( natural W/3 experiment* ) OR TITLE-ABS-KEY ( naturalistic W/3 experiment* ) ) ) AND ( ( TITLE-ABS-KEY ( population W/3 health ) OR TITLE-ABS-KEY ( public W/3 health ) ) ) AND ( EXCLUDE ( PUBYEAR , 1991 ) ) AND ( EXCLUDE ( SRCTYPE , "b" ) ) AND ( EXCLUDE ( DOCTYPE , "re" ) OR EXCLUDE ( DOCTYPE , "cp" ) OR EXCLUDE ( DOCTYPE , "ed" ) OR EXCLUDE ( DOCTYPE , "no" ) )
