# Supplementary material for: Using natural experiments to improve public health evidence: a review of context and utility for obesity prevention
Source: Health Res Policy Syst. 2020 May 18;18:48. doi: 10.1186/s12961-020-00564-2 (PMC7236508; doi:10.1186/s12961-020-00564-2)
Supplement: Supplementary file 2 — Additional file 2: Table S2. Describes studies excluded through abstract review. [file 12961_2020_564_MOESM2_ESM.docx]

## Additional file 2

### Table S1. Studies excluded through abstract review

| **Study (first author, year citation)*** | **Exclusion reason** |
| --- | --- |
| Anderson P, 2011 [1] | commentary or opinion |
| Arendt, JN, 2005 [2] | not PANO (education and general health) |
| Astell-Burt, 2015 [3] | not PANO (mental distress related to neighbourhood crime |
| Astell-Burt, 2015 [4] | not PANO (mental distress related to socioeconomic status) |
| Berrigan D, 2010 [5] | does not identify as natural experiment |
| Brown V, 2015 [6] | review or meta-analysis |
| Brownson RC, 2010 [7] | commentary or opinion |
| Copeland JL 2017 [8] | does not identify as natural experiment |
| Coutts C, 2010 [9] | not PANO (green space access and mortality) |
| Coyle E, 2009 [10] | commentary or opinion |
| Dickerson J, 2016 [11] | protocol, method or tool |
| Engler-Stringer, 2016 [12] | protocol, method or tool |
| Heinen E, 2015 [13] | does not identify as natural experiment |
| Huang TTK, 2016 [14] | protocol, method or tool |
| Hutter H, 2016 [15] | not PANO (exposure to plastic products and urinary bisphenol A) |
| Johnson F, 2011 [16] | review or meta-analysis |
| Kaspersen S, 2017 [17] | not PANO (economic risk of downsizing and pharmaceutical usage) |
| Kim S, 2014 [18] | not PANO (malnutrition) |
| Klimek P, 2014 [19] | review or meta-analysis |
| Lawlor DA, 2016 [19] | commentary or opinion |
| Lindeboom M, 2010 [20] | not PANO (malnutrition) |
| Ljungdahl S, 2015 [21] | review or meta-analysis |
| McIntyre L, 2017 [22] | not PANO (food insecurity and home ownership) |
| McKay JA, 2016 [23] | review or meta-analysis |
| O'Flaherty M, 2016 [24] | commentary or opinion |
| Ogilvie D, 2012 [25] | protocol, method or tool |
| Pearce JR, 2011 [26] | review or meta-analysis |
| Peng X, 2016 [27] | not PANO (malnutrition) |
| Petticrew M, 2005 [28] | commentary or opinion |
| Piperata B, 2016 [29] | not PANO (welfare payments and food security) |
| Schaller A, 2016 [30] | protocol, method or tool |
| Scharff R, 2016 [31] | not PANO (foodborne disease surveillance) |
| Silveirinha de Oliveira E, 2013 [32] | protocol, method or tool |
| Thern 2017 [33] | not PANO (alcohol exposure and deaths) |
| Tully MA, 2013 [34] | protocol, method or tool |
| Veugelers P, 2008 [35] | does not identify as natural experiment |
| Wang Y, 2015 [36] | review or meta-analysis |
| Wolch JR, 2014 [37] | review or meta-analysis |
| Yu CY, 2017 [37] | not PANO (economic benefits of built environment design) |

Abbreviation: PANO= physical activity-, nutrition- or obesity-related.

### *References for Table S1.

1. Anderson P, Gual A. Reflections on science and the governance of alcohol policy. Addiction. 2011;106:67-70.

2. Arendt JN. Does education cause better health? A panel data analysis using school reforms for identification. Economics of Education Review. 2005;24(2):149-60.

3. Astell-Burt T, Feng X. Investigating 'place effects' on mental health: implications for population-based studies in psychiatry. Epidemiology and Psychiatric Sciences. 2015;24(1):27-37.

4. Astell-Burt T, Feng X, Kolt GS, Jalaludin B. Does rising crime lead to increasing distress? Longitudinal analysis of a natural experiment with dynamic objective neighbourhood measures. Social Science & Medicine. 2015;138:68-73.

5. Berrigan D, Pickle LW, Dill J. Associations between street connectivity and active transportation. International Journal of Health Geographics. 2010;9.

6. Brown V, Moodie M, Carter R. Congestion pricing and active transport - evidence from five opportunities for natural experiment. Journal of Transport & Health. 2015;2(4):568-79.

7. Brownson RC, Chriqui JF, Burgeson CR, Fisher MC, Ness RB. Translating epidemiology into policy to prevent childhood obesity: The case for promoting physical activity in school settings. Annals of Epidemiology. 2010;20(6):436-44.

8. Copeland JL, Currie C, Walker A, Mason E, Willoughby TN, Amson A. Fitness Equipment in Public Parks: Frequency of Use and Community Perceptions in a Small Urban Centre. Journal of Physical Activity & Health. 2017;14(5):344-52.

9. Coutts C, Horner M, Chapin T. Using geographical information system to model the effects of green space accessibility on mortality in Florida. Geocarto International. 2010;25(6):471-84.

10. Coyle E, Huws D, Monaghan S, Roddy G, Seery B, Staats P, et al. Transport and health - a five-country perspective. Public Health. 2009;123(1):e21-e3.

11. Dickerson J, Bird PK, McEachan RRC, Pickett KE, Waiblinger D, Uphoff E, et al. Born in Bradford's Better Start: an experimental birth cohort study to evaluate the impact of early life interventions. Bmc Public Health. 2016;16.

12. Engler-Stringer R, Muhajarine N, Ridalls T, Abonyi S, Vatanparast H, Whiting S, et al. The Good Food Junction: a Community-Based Food Store Intervention to Address Nutritional Health Inequities. Jmir Research Protocols. 2016;5(2).

13. Heinen E, Panter J, Dalton A, Jones A, Ogilvie D. Sociospatial patterning of the use of new transport infrastructure: Walking, cycling and bus travel on the Cambridgeshire guided busway. Journal of Transport & Health. 2015;2(2):199-211.

14. Huang TTK, Wyka KE, Ferris EB, Gardner J, Evenson KR, Tripathi D, et al. The Physical Activity and Redesigned Community Spaces (PARCS) Study: Protocol of a natural experiment to investigate the impact of citywide park redesign and renovation. Bmc Public Health. 2016;16.

15. Hutter HP, Kundi M, Hohenblum P, Scharf S, Shelton JF, Piegler K, et al. Life without plastic: A family experiment and biomonitoring study. Environmental Research. 2016;150:639-44.

16. Johnson F, Mavrogianni A, Ucci M, Vidal-Puig A, Wardle J. Could increased time spent in a thermal comfort zone contribute to population increases in obesity? Obesity Reviews. 2011;12(7):543-51.

17. Kaspersen SL, Pape K, Carlsen F, Ose SO, Bjorngaard JH. Employees' drug purchases before and after organizational downsizing: a natural experiment on the Norwegian working population (2004-2012). Scandinavian Journal of Work Environment & Health. 2017;43(4):307-15.

18. Kim S, Deng Q, Fleisher BM, Li S. The lasting impact of parental early life malnutrition on their offspring: Evidence from the China great leap forward famine. World Development. 2014;54:232-42.

19. Lawlor DA, Tilling K, Smith GD. Triangulation in aetiological epidemiology. International Journal of Epidemiology. 2016;45(6):1866-86.

20. Lindeboom M, Portrait F, van den Berg GJ. Long-run effects on longevity of a nutritional shock early in life: The Dutch Potato famine of 1846-1847. Journal of Health Economics. 2010;29(5):617-29.

21. Ljungdahl S, Bremberg SG. Might extended education decrease inequalities in health? - A meta-analysis. European Journal of Public Health. 2015;25(4):587-92.

22. McIntyre L, Wu X, Kwok C, Emery JCH. A natural experimental study of the protective effect of home ownership on household food insecurity in Canada before and after a recession (2008-2009). Canadian Journal of Public Health. 2017;108(2):e135-e44.

23. McKay JA, Mathers JC. Maternal folate deficiency and metabolic dysfunction in offspring. Proceedings of the Nutrition Society. 2016;75(1):90-5.

24. O'Flaherty M, Guzman M. Keeping Public Health Clean: Food Policy Barriers and Opportunities in the Era of the Industrial Epidemics. Aims Public Health. 2016;3(2):228-34.

25. Ogilvie D, Bull F, Cooper A, Rutter H, Adams E, Brand C, et al. Evaluating the travel, physical activity and carbon impacts of a 'natural experiment' in the provision of new walking and cycling infrastructure: methods for the core module of the iConnect study. Bmj Open. 2012;2(1).

26. Pearce JR, Maddison R. Do enhancements to the urban built environment improve physical activity levels among socially disadvantaged populations? International Journal for Equity in Health. 2011;10.

27. Peng X, Conley D. The implication of health insurance for child development and maternal nutrition: evidence from China. European Journal of Health Economics. 2016;17(5):521-34.

28. Petticrew M, Cummins S, Ferrell C, Findlay A, Higgins C, Hoy C, et al. Natural experiments: An underused tool for public health? Public Health. 2005;119(9):751-7.

29. Piperata BA, McSweeney K, Murrieta RS. Conditional Cash Transfers, Food Security, and Health: Biocultural Insights for Poverty-Alleviation Policy from the Brazilian Amazon. Current Anthropology. 2016;57(6):806-26.

30. Schaller A, Dejonghe L, Alayli-Goebbels A, Biallas B, Froboese I. Promoting physical activity and health literacy: study protocol for a longitudinal, mixed methods evaluation of a cross-provider workplace-related intervention in Germany (The AtRisk study). Bmc Public Health. 2016;16.

31. Scharff RL, Besser J, Sharp DJ, Jones TF, Peter GS, Hedberg CW. An Economic Evaluation of PulseNet: A Network for Foodborne Disease Surveillance. American Journal of Preventive Medicine. 2016;50(5):S66-S73.

32. Silveirinha de Oliveira E, Aspinall P, Briggs A, Cummins S, Leyland AH, Mitchell R, et al. How effective is the Forestry Commission Scotland's woodland improvement programme--'Woods In and Around Towns' (WIAT)--at improving psychological well-being in deprived urban communities? A quasi-experimental study. BMJ open. 2013;3(8):e003648-e.

33. Thern E, Jia T, Willmer M, de Munter J, Norstrom T, Ramstedt M, et al. No effects of increased alcohol availability during adolescence on alcohol-related morbidity and mortality during four decades: a natural experiment. Journal of Epidemiology and Community Health. 2017;71(11):1072-7.

34. Tully MA, Hunter RF, McAneney H, Cupples ME, Donnelly M, Ellis G, et al. Physical activity and the rejuvenation of Connswater (PARC study): Protocol for a natural experiment investigating the impact of urban regeneration on public health. BMC Public Health. 2013;13(1).

35. Veugelers P, Sithole F, Zhang S, Muhajarine N. Neighborhood characteristics in relation to diet, physical activity and overweight of Canadian children. International Journal of Pediatric Obesity. 2008;3(3):152-9.

36. Wolch JR, Byrne J, Newell JP. Urban green space, public health, and environmental justice: The challenge of making cities 'just green enough'. Landscape and Urban Planning. 2014;125:234-44.

37. Yu CY, Xu M, Towne SD, Iman S. Assessing the economic benefits and resilience of complete streets in Orlando, FL: A natural experimental design approach. Journal of Transport and Health. 2017.
