# Supplementary material for: Using natural experiments to improve public health evidence: a review of context and utility for obesity prevention
Source: Health Res Policy Syst. 2020 May 18;18:48. doi: 10.1186/s12961-020-00564-2 (PMC7236508; doi:10.1186/s12961-020-00564-2)
Supplement: Supplementary file 3 — Additional file 3: Figure S1. Showing the use of natural experiment studies surrounding nutrition and physical activity interventions on obesity. [file 12961_2020_564_MOESM3_ESM.docx]

## Additional file 3

### Figure S1. Use of natural experiment studies surrounding nutrition and physical activity interventions on obesity, 1997-2017.
