# Supplementary material for: Using natural experiments to improve public health evidence: a review of context and utility for obesity prevention
Source: Health Res Policy Syst. 2020 May 18;18:48. doi: 10.1186/s12961-020-00564-2 (PMC7236508; doi:10.1186/s12961-020-00564-2)
Supplement: Supplementary file 4 — Additional file 4: Table S3. Describes the research aims of included studies. [file 12961_2020_564_MOESM4_ESM.docx]

## Additional file 4

### Table S2. Research aims of included studies.

| **First author, year*** | **Research aim** | **Purpose** |
| --- | --- | --- |
| Allais O, 2015 | to evaluate and compare front-of-pack nutrition labels and nutritional taxes | policy impact |
| Barlow P, 2017 | to study the effect of withdrawal of tariffs on high fructose corn syrup products on consumption of the same in Canada | policy impact |
| Barradas SC, 2017 | to explore the difference of quality of life and life satisfaction perceptions between participants and non-participants | intervention effectiveness |
| Bernatchez AC, 2015 | to establish correlation between awareness of public bike share program over time and docking stations distance from residence | intervention effectiveness |
| Brandt EJ, 2017 | to determine whether trans-fatty acid restrictions in counties were associated with fewer hospital admissions for myocardial infarction and stroke compared with counties without restrictions | policy impact |
| Cawley J, 2007 | to examine the impact of state PE requirements on student PE exercise time; to estimate the causal impact of PE on overall student physical activity and weight | policy impact |
| Cawley J, 2010 | to determine effect of income (social security) on weight | assess health inequalities in the population |
| Christian H, 2013 | to examine whether people moving into a ‘liveable neighbourhood’ housing development engaged in more walking than people who move to other types of developments; to examine differences in the built environment features of development types and changes in walking | assess environmental determinants of health in the population |
| Christian H, 2017 | to investigate longitudinal associations between objective and perceived neighbourhood environment measures and neighbourhood recreational walking | assess environmental determinants of health in the population |
| Cohen DA, 2012 | to determine how well the fitness equipment is used after installation; who uses it, frequency of use and how; whether parks with new equipment have more users and whether users more physically active in these parks than before the equipment was installed; and the cost-effectiveness of the equipment, based upon physical activity. | assess environmental determinants of health in the population |
| Copeland JL, 2017 | to examine how frequently active park equipment is used in a small urban centre; if active park fitness equipment attracts adults to parks; if physical activity intensity was higher among those who used the fitness equipment as compared with those who used parks in other ways; and garner community perceptions | Intervention effectiveness |
| Cornelsen L, 2017 | to assess whether the implementation of the levy in combination with non-price activities is associated with changes in sales of non-alcoholic beverages | Policy impact |
| Cranney L, 2016 | to assess the impact of the installation and promotion of an outdoor gym in a park setting on physical activity levels of park users and to examine the characteristics of outdoor gym users, motivators, enablers and barriers to use. | Intervention effectiveness |
| Datar A, 2017 | to examine whether state competitive food and beverage policies were associated with children’s BMI, overweight or obesity | Policy impact |
| Dill J, 2014 | to evaluate changes in physical activity and active transportation associated with installation of new bicycle boulevards | Intervention effectiveness |
| Dubowitz T, 2015 | to evaluate the extent to which a major change in the food environment (access to healthy food outlet) of a low-income, predominantly Black neighbourhood influences change in residents’ diet and food purchasing | assess social determinants of health in the population |
| Elbel B, 2011 | to examine whether calorie labels help inform parent/caregiver and adolescent food choices | Policy impact |
| Ferguson M, 2017 | to examine impact of pricing strategies on food and beverage sales, perceived level of success, key enablers and barriers to implementation, and perceived benefits associated with pricing strategies | intervention effectiveness |
| Frew EJ, 2014 | to determine the cost-effectiveness of a physical activity programme | intervention effectiveness |
| Fu H, 2012 | to ascertain what effects migration has on obesity risks among Vietnamese immigrants | assess social determinants of health in the population |
| Gee GC, 2015 | to evaluate whether migrants and non- migrants differ in BMI, waist circumference or waist-to- hip ratio | assess social determinants of health in the population |
| Giles-Corti B, 2013 | to examine the impact of the built environment on walking for transport and recreation following relocation to 'new urbanism' inspired neighbourhoods | assess environmental determinants of health in the population |
| Hobin E, 2017 | to examine the extent to which consumers respond to an on-shelf nutrition labelling system in supermarkets | Intervention effectiveness |
| Jancey JM, 2016 | to determine the impact of relocating to new active design office building on office workers’ sedentariness and level of physical activity | assess environment changes on population health behaviour |
| Jones-Smith JC, 2017 | to examine whether casinos are associated with obesity-related health in utero | assess social determinants of health in the population |
| Jürges, H, 2011 | to investigate the causal effect of schooling on health behaviour such as smoking and related outcomes such as obesity | assess social determinants of health in the population |
| Kapinos KA, 2017 | to establish causal evidence on the role of Caesarean delivery on maternal postpartum weight | Intervention effectiveness |
| Kesten JM, 2014 | to understand the nature of new transport infrastructure and how it was experienced | Intervention effectiveness |
| Kodish SR, 2016 | to understand the perceived pathways by which casinos impact individual and community health through voices of the community | assess social determinants of health in the population |
| Lee C, 2017 | to understand the causal relationships between environmental improvements and mode shifts; specifically, whether changes in home-to-school travel environments lead to mode shifts from sedentary to active | assess environmental determinants of health in the population |
| Madsen KA, 2011 | to assess the impact of BMI screening with parental notification on weight status for public school students | Intervention effectiveness |
| Odoms-Young AM, 2014 | to assess the impact of a food packages mandated by the Special Supplemental Nutrition Program on dietary intake and home food availability in low-income African-American and Hispanic parent/child dyads | Intervention effectiveness |
| Ovrum A, 2014 | To assess impacts of a nationwide Norwegian School Fruit Scheme on fruit intake | Intervention effectiveness |
| Pollack CE, 2014 | to assess whether type of public housing (scattered or clustered) is associated with the perceived health and health behaviours of residents’ social networks | assess social determinants of health in the population |
| Ram B, 2016 | to establish whether physical activity and other health behaviours show sustained changes among individuals and families relocating to new social and built environment with active design features | Intervention effectiveness |
| Sadler RC, 2013 | whether a neighbourhood-level food retail intervention affects dietary habits or food security | Intervention effectiveness |
| Schultz CL, 2017 | to evaluate the impact of street crossing infrastructure modifications on park use and park-based physical activity in a low-income and African American community | Intervention effectiveness |
| Simões EJ, 2017 | to evaluate impact of community PA program on population levels of LTPA and associations of LTPA with self-reported participation in and knowledge of program | Intervention effectiveness |
| Stanley K, 2016 | to understand daily travel patterns and the impact of particular interventions or disruptions on travel | assess environment changes on population health behaviour |
| Stone MR, 2012 | to evaluate school board policy for daily PA through measuring proportion of children who participate in such, and the proportion who managed to achieve sustained MVPA within these sessions | Policy impact |
| Sutherland LA, 2010 | to examine the effect of a comprehensive storewide supermarket point-of-purchase nutrition navigation intervention by using a shelf-label 3-tiered star icon on consumer food and beverage choices and their associated nutritional quality | Intervention effectiveness |
| Torres A, 2017 | to assess the effectiveness of the community PA program in increasing PA among users of nine parks | Intervention effectiveness |
| Tudor-Locke C, 2008 | to investigate the extent and pattern of tracking of pedometer-determined physical activity in a persons who moved to 'new urbanism' inspired neighbourhoods | assess environment changes on population health behaviour |
| Veitch J, 2012 | to examine whether improvements to a park increased its use and park-based physical activity of users | assess environment changes on population health behaviour |
| Wagner B, 2013 | to estimate environmental effects of the correlation between context of physical activity and alcohol consumption among college students unbiased by gene-environment correlations | assess environmental determinants of health in the population |
| Watson B, 2016 | to examine whether increasing economic insecurity causes weight gain and obesity | assess social determinants of health in the population |
| Zick CD, 2014 | to assess whether day light savings time is associated with increased time spent in MVPA | assess environment changes on population health behaviour |

Table legend: Abbreviations: BMI = body mass index, LTPA = leisure-time physical activity, MVPA = moderate to vigorous physical activity, PA = physical activity, PE = physical education.

*Full references for these studies are included in the main manuscript.
